# A System Dynamics Model of Caries Preventive Interventions in Thailand’s School-Aged Population

**DOI:** 10.3290/j.ohpd.b4721201

**Published:** 2023-12-04

**Authors:** Tin Htet Oo, Sukanya Tianviwat, Songchai Thitasomakul, Phongpat Sontamino

**Affiliations:** a Graduate Student, Department of Preventive Dentistry, Faculty of Dentistry, Prince of Songkla University, Songkhla, Thailand. Concept and design, conducted the study in partial fulfillment of requirments for PhD degree, wrote the manuscript.; b Associate Professor, Evidence-Based Dentistry for Oral Health Care and Promotion Phase II Research Unit, Department of Preventive Dentistry, Faculty of Dentistry, Prince of Songkla University, Songkhla, Thailand. Idea, hypothesis, supervised the study, concept and design, data manipulation and analysis, critically revised the manuscript.; c Associate Professor, Evidence-Based Dentistry for Oral Health Care and Promotion Phase II Research Unit, Department of Preventive Dentistry, Faculty of Dentistry, Prince of Songkla University, Songkhla, Thailand. Contributed substantially to study concept and design, data analysis, critically revised the manuscript.; d Assistant Professor, Department of Mining and Materials Engineering, Faculty of Engineering, Prince of Songkla University, Songkhla, Thailand. Contributed substantially to study concept, design and data analysis, critically revised the manuscript.

**Keywords:** dental caries, preventive intervention, system dynamics model

## Abstract

**Purpose:**

To compare the long-term effects of the Ministry of Public Health’s (MOPH) caries preventive interventions for 6- to 12-year-olds (supervised toothbrushing [STB], dental sealant, and combined STB+sealant) to the base case (no intervention) using the System Dynamics Model.

**Materials and Methods:**

The System Dynamics Model was used to evaluate the intervention scenarios of supervised toothbrushing (STB), sealant, and combined STB+sealant with the base-case scenario. The effectiveness data for the model’s interventions were obtained from systematic reviews and meta-analyses.

**Results:**

The model determined that the caries-free population increased by 36.2%, 25.5%, and 14.5%, while the caries-affected population decreased by 8.1%, 5.5%, and 3.1% in the combined STB+sealant, sealant, and supervised toothbrushing scenarios compared to the base case at 15 years of age.

**Conclusion:**

Combined STB+sealant is the most efficacious intervention among those administered to children between the ages of 6 and 12 with permanent teeth. In addition, the System Dynamics Model could be helpful in comparing interventions or policies to determine the optimal intervention for a given population.

Although dental caries is preventable, it is still an oral disease with a high societal burden not only in Thailand but globally as well.^24^ In developed nations, studies indicate that the prevalence of caries has decreased over the past several decades.^10,23^ Nonetheless, some studies have documented an upward trend in caries prevalence in a number of developing nations.^2,23^ The high prevalence of caries in developing nations is due to poor oral hygiene practices, low awareness of caries, an increase in sugar consumption, inadequate access to dental health prevention programmes, and inadequate fluoride exposure.^23^ According to the 8th National Oral Health Survey (NOHS) conducted in Thailand in 2017, the prevalence of caries among 12- and 15-year-olds remained high at 52.0% and 62.7%, respectively.^5^ People with caries may experience pain, nutritional imbalance, aesthetic and functional issues.^20^ In addition, tooth loss caused by caries was the most prevalent consequence of caries.^21^

Caries prevention is essential due to the high prevalence of caries. As a result, various preventive programmes, such as oral health education, toothbrushing, fluoride varnish, and application of dental sealants, have been designed to address caries both in individuals and communities.^6,7,8,30^ In Thailand, the Ministry of Public Health (MOPH) proposed preventive oral health programmes for schoolchildren, including toothbrushing with fluoride toothpaste and applying dental sealants.^4^ Supervised toothbrushing (STB) and dental sealant preventive programmes were proposed for school children ages 6 to 12 by experts from the Ministry of Public Health and the Ministry of Education, Thailand. in order to develop personal skills, establish health policy, and reorient health services in accordance with the Ottawa Charter.^4^

However, preventive interventions for improved decision-making and an alternative programme are required. Models of simulation would help policymakers in planning long-term processes through decision-making.^17^ The Markov Model, System Dynamics Model (SDM), Microsimulation Model, and Decision-tree Model were cited in studies as dependable caries interventions.^26^ The System Dynamics Model (SDM) is a computer simulation that assists stakeholders in determining the most beneficial policy for the community.^18^ Since the 1970s, it has been applied to health issues such as disease epidemiology, an economical approach in health care interventions, transfers of patients in emergency and extended care, and caries interventions in dental care.^14,26^ In contrast to the Markov and microsimulation models, which describe the transition of health states over time and repeated events, SDM demonstrates the interaction between entities of the system by modeling the rate of change of the system and enables the visualisation of feedback loops that arise from the interaction.^28,33^ It should be approached as a cohort-based simulation model under both individual preventive interventions and combinations of interventions. In this study, SDM was investigated for caries preventive interventions envisioned by the Ministry of Public Health (MOPH), including supervised STB, sealant application, and their combination. The interventions were primary preventive interventions for caries, as they focused on preventing the onset of disease in a healthy state.^34^

Therefore, the objective of the study was to estimate the caries outcome given caries preventive interventions provided to 6- to 12-year-olds by the Ministry of Public Health (MOPH); the preventive interventions comprised STB, sealant application, and combined STB+sealant. The estimated caries outcome was compared with the no-interventions scenario (base case) by conducting the SDM.

## Methodology

The methodology can be divided into two sections. The first section consisted of analysing systematic reviews and meta-analyses of interventions to determine the effectiveness of interventions performed by researchers; these results were input into the SDM as parameters. The second section consisted of conducting the SDM to estimate caries prevention outcomes. Group model building (GMB) was used to construct the SDM in order to define the model structure, identify the database used for the model, and validate the model. GMB is designed to improve the robustness of the model.

### Systematic Review and Meta-Analysis

The effects of STB and sealants (implemented in 6- to 12-year-olds) on caries were identified by systematic review and meta-analyses.^22^ Studies on STB were searched in Cochrane, PubMed, Web of Science, and SCOPUS databases.^22^ The included studies were identified according to PICO (Participants, Intervention, Comparison, and Outcome).^12^ The protocol was registered with PROSPERO (CRD42022376887). The process followed the guidelines of the systematic review of interventions provided in the Cochrane Handbook, version 5.1.0.^12^ For sealants, the studies were retrieved from a previous, recent review.^1^ Meta-analyses with the random-effect model for both interventions were performed using RevMan 5.3 software.^22^ The estimated effect size in the meta-analysis was risk ratio (RR) and efficacy rates of interventions were calculated by the formula ([1-RR]*100) to enter in the model (supplementary Table 1).

**Supplementary Table 1:** Search terms for studies

No		Interventions		Participants		Outcome
1.	Supervised toothbrushing for 6- to 15-year-olds	(“Toothbrushing” [Mesh] OR “Education, Dental” [Mesh])	AND	(“Child” [Mesh] OR “Schools” [Mesh])	AND	(“Dental caries” [Mesh])
2.	Sealant for 6- to 15-year-olds	(“Pit and Fissure Sealants” [Mesh] OR “Dental Sealant”)	AND	(“Child” [Mesh] OR “Schools” [Mesh])	AND	(“Dental caries” [Mesh])

### Group Model Building (GMB)

The modeling for this investigation focused on the age of permanent dentition. The final model was determined through three sessions of group model building (GMB) involving researchers and experts in related fields. SDM specialists and experts with valuable knowledge and at least ten years of experience working with adolescent populations were selected. Experts from the Royal College of Dental Surgeons of Thailand (3), the Ministry of Public Health (6), Provincial Health Authorities (7), central and community hospitals (7), and leading universities and colleges of public health (7) were all involved.

GMB sessions included determining the time horizon, identifying the key variables and their behaviours, devising the causal loop diagram (CLD), developing the stocks and flows diagram, and validating the model. In GMB 1, the researcher guided participants in identifying the time horizon, prospective variables associated with the model’s outcomes, and the frequency with which variables would change over time based on various scenarios. The participants then discussed the natural history and progression of disease states in response to the initial question: “What are the states of diseases, including treated states, and how do they relate?” in addition to “How does the intervention affect the disease?” Then, the causal loop diagram was constructed and modified after participants consulted to adjust variables. In order to quantitatively estimate the outcomes, the CLD was converted into a stocks and flows diagram following participant consensus. In addition, participants were asked which database should be used to retrieve the necessary parameter values. In GMB 2, the model was then validated by experts, as described in the following section. In the final session, the model was finalised by discussing the simulation results in various scenarios and soliciting feedback.

### Model Structure

The System Dynamics Model (SDM) involves two parts: qualitative and quantitative. The qualitative part showed the causal relationship of caries-related events and identified the feedback loops among them by a CLD. The quantitative part translated the CLD for evaluating the conditions quantitatively represented as stocks and flows diagram.

**Fig 1:**
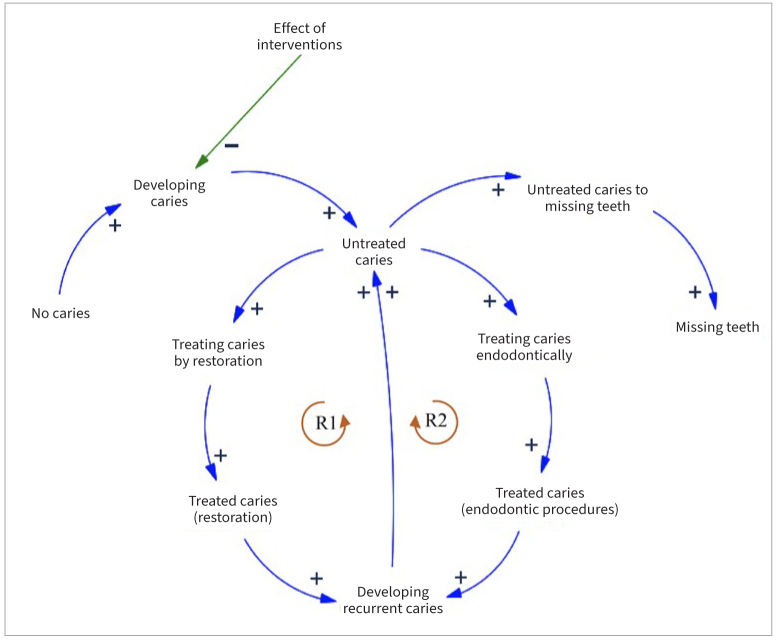
Causal loop diagram (CLD).

The CLD presents the causal relationship of no caries, caries, related events of caries, and feedback loops among them (Fig 1). A positive sign (+) in the diagram means that a variable adds to another one and a negative sign (-) that it subtracts from the other.^9^ An increase in no caries is associated with an increase in developing caries, an increase in developing caries is associated with an increase in untreated caries and an increase in untreated caries is associated with an increase in tooth loss. There are two reinforcing loops (R1 and R2) in the diagram. If variables continue to move in the same direction, it is a reinforcing loop.^9^ R1 indicates that an increase in untreated caries is associated with an increase in treated caries (restoration) and recurrent caries resulting from restoration. R2 also indicates that an increase in untreated caries is associated with an increase in treated caries (using endodontic procedures) and the development of recurrent caries after endodontic treatment. An increase in recurrent caries from cases that have been treated results in an increase in untreated caries. When the intervention is implemented, it will reduce caries development and its consequences: untreated caries, treated cases, recurrent caries from treated cases, and tooth loss.

**Fig 2:**
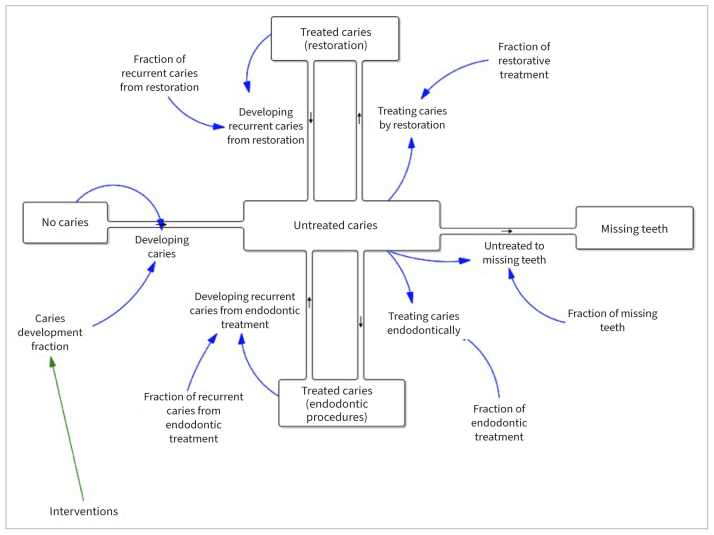
Stocks and flows diagram.

CLD was translated into the stocks and flows diagram, shown in Fig 2, to evaluate the outcomes quantitatively. The square blocks in the diagram are denoted as stocks and the arrows represent the flows. The stocks are defined as states or levels of variables moving through the system.^17^ The flows control the movement of stocks or determine the changes in the stocks by in-and-out valves of the stocks.^17^ The quantitative outcomes are attributed to the stocks in the model. The fractions affect the rate of flows: for example, the caries development fraction influences the rate of developing caries, and the fraction of restorative treatment affects the rate of treating caries with restorations. It is assumed that when the intervention is set, the interventions could reduce the caries development fraction, tending to slow the rate of developing caries (Fig 2).

Vensim DSS version 6.4 software was used for running the model. The model simulated the Thai population born in 2021 from 6 to 15 years old. The total population, based on Thailand’s National Statistical Office, was 678,243. The results are presented when the children are secondary–school-aged, i.e., 15 years old, and the interventions are discontinued at this age.

### Source of Data

The efficacy rates of the interventions to be used in the model were obtained from systematic reviews and meta-analyses.^22^ The other required parameters in the model were obtained from Thailand’s data: the 8th National Oral Health Survey (NOHS), the Health Data Center (HDC), the National Statistical Office, literature, and experts’ opinions. Data used in the model were validated by experts in group model building (GMB) sessions (supplementary Table 2).

**Supplementary Table 2:** Efficacy rates for the interventions

Interventions	Effect estimate, Risk Ratio (RR) from the meta-analysis	Efficacy rates (1-RR) x 100%	Coverage rates	Efficacy rates after adjusted with coverage rates #	Assumption
Supervised toothbrushing (STB)	0.9 [22]	1- 0.9 x 100% = 10% [22]^A^	92.5% (4,194,000/ 4,534,544 x 100%) [15]^B^	0.1 x 92.5% = 9.25%	The efficacy rate of STB in 95% coverage rate is 10% (due to the not full coverage) and STB can reduce developing caries by 9.25% per year.
Sealant	0.29 [22]	1- 0.29 x 100% = 71% on molar teeth (58% for all teeth based on experts’ opinions) [22]^A^	27% (1,443,484/ 5,369,451 x 100%) [11]^C^	0.58 x 27% = 15.7%	According to meta-analysis, sealant has a 71% caries reduction on molar teeth. Based on experts’ opinions using their available existing data, sealant was regarded with a 58% caries reduction for all teeth [i.e. caries % for molars is 82%, for other teeth 18%, and 0% for caries prevention on other teeth. The efficacy rate on molars is 58% (82% x 0.71) and for other teeth 0% (18% x 0). The efficacy rate for all teeth is 58% (58% + 0%)]. The efficacy rate of sealant with a 27% coverage rate is 15.7% (due to incomplete coverage) and sealant can reduce developing caries by 15.7% per year.
Combined STB+Sealant	–	60% (based on experts’ opinions) [22]	33% (149,827/ 452,860 x 100%) [15]^B^	0.60 x 33% = 19.8%	According to each efficacy rate of STB and sealant and experts’ opinions based on their existing data, the efficacy rate of combined STB+Sealant was determined as 60%. The efficacy rate of combined STB+Sealant in 33% coverage rate was 19.8% (due to incomplete coverage) and STB+Sealant can reduce caries development by 19.8% per year.

^A^Efficacy rate based on a meta-analysis of intervention. ^B^Coverage rate based on evaluation of oral health promotion and prevention in school children project under National Health Security. ^C^ Coverage rate based on Health Data Center, a national database consisting of Ministry of Public Health medical and health information. ^#^Efficacy rates after adjusted with coverage rates = efficacy rate x coverage rate.

### Model Validation

The structural validity of the model was conducted by participants in the GMB sessions. As a structure-based validation, the adequacy of the model boundary, model’s structures, verification of parameters, and dimensional consistency of the model’s equations were examined.^25^ To check the model’s boundary adequacy, caries and related issues as well as setting interventions were examined to deterimine whether they were consistent with the conceptual framework and objective of the study. The states of caries were verified if it was relevant to the natural history of the disease as the model’s structure verification. For the model’s parameters verification, they were checked whether they were relevant to the information of the existing system. The equations of the model were examined to determine whether they dimensionally corresponded to the real system. An agreement was made between the experts that the structure of the model accurately reflects the real system.

A behaviour replication test was performed for the behavioural validity of the model.^17^ The simulated behaviour of the variables (dental with caries-related events) was compared to national historical reference data in which the interventions were implemented in order to determine whether it was consistent with the reference data.

### Scenario Analysis

The interventions were analysed as intervention scenarios comparing the base-case scenario as follows. Effectiveness of intervention means achieving the caries preventive effect of intervention among the intended population by setting the intervention based on a desired policy. It is assumed that the interventions (STB, sealant, and STB+sealant) were provided from the age of 6 to 12 years, as the Ministry of Public Health (MOPH) launched the preventive programmes for school ages.^4^ It is assumed that the system covers these ages, since the children in these age ranges are primary-school children, and are the chief recipients of caries preventive programmes.^6,8,19^ The efficacy rates of the interventions were retrieved from meta-analyses by the formula ([1-RR]*100%) and were adjusted with coverage rates in Thailand and input into the model. The coverage rate of the intervention refers to the proportion of the population who received the interventions in Thailand.

#### Base case

The base-case scenario refers to the situation in which no intervention was given. It means that the policy did not cover the target population to achieve the desired effect of the intervention. The caries development rate under the base-case scenario set in the model was retrieved as the rate of developing caries per year without the effect of interventions.^31^ It served as a reference point for comparing intervention scenarios where caries development was reduced due to the effect of interventions as mentioned below. All model parameters in the base case-scenario remained unchanged over the simulation run.

#### Supervised toothbrushing (STB)

STB aims to prevent the development of caries using the correct toothbrushing technique, along with fluoride toothpaste and appropriate toothbrush design, twice daily under the supervision of dental health professionals or trained teachers or parents.^4,8^ The coverage rate of the intervention among 6- to 12-year-olds in Thailand was 92.5%.^15^ The efficacy rate for this age group was retrieved from the meta-analysis as 10%.^22^ It was adjusted with the coverage rate by multiplying with the coverage rate 92.5% (0.1 x 92.5%), and the adjusted efficacy rate 9.25% was set in the model intended to reduce the caries development rate (supplementary Table 2).

#### Dental Sealant

The application of resin-based sealants in deep pits and fissures of permanent teeth by dental professionals is intended to prevent caries.^6^ In Thailand, the coverage rate of sealants among 6- to 12-year-olds was 27%.^11^ According to a meta-analysis, dental sealants have provide a 71% caries reduction rate on molars in this age group.^22^ Based on that meta-analysis and experts’ opinions using their available existing data, dental sealants were thought to provide a 58% decreased risk of caries for all teeth.^22^ It was multiplied by the coverage rate of 27% (0.58 x 27%), and the adjusted efficacy rate of 15.7% was set in the model to decrease the rate of caries development (Supplementary Table 2).

#### Combined supervised toothbrushing and sealant (STB+Sealant)

The descriptions of STB and sealant are the same as mentioned above. The coverage rate of the combined STB+sealant in Thailand among 6- to 12-year-olds was 33%.^15^ The efficacy rates of STB and sealant before adjusting with the coverage rates were 10% and 58%, respectively, as mentioned above. According to each rate and experts’ opinions based on their existing data, the efficacy rate of combined intervention was determined as 60%.^22^ After adjusting the coverage rate to 33% (0.6 x 33%), the adjusted efficacy rate of 19.8% was set in the model to slow the caries development rate (Supplementary Table 2).

### Sensitivity Analysis

Sensitivity analysis was done as multivariate sensitivity analysis with random uniform distribution in Vensim DSS version 6.4 software (Ventana Systems; Harvard, MA, USA) to examine how a change in the parameters would influence the outcomes. The initial parameter values for the stocks and fractions that affect the rate of flows were entered into the model. The values of fractions were varied by ±10%. This figure came from the change in children’s coverage compared with and without the national policy of comprehensive care among children.^3^

### Outcomes of the Model

The stocks in the model defined the main outcomes: no caries, caries, and consequences of caries as follows:

- Population with no caries
- Population with untreated caries
- Population with restorations
- Population with endodontic treatment
- Population with missing teeth

### Ethical Considerations

This study received ethical approval from the Human Research Ethics Committee of the Faculty of Dentistry, Prince of Songkla University, EC6503-011 on 7 March 2022.

## Results

As shown in Table 1, the number of 15-year-olds with no caries under combined STB+sealant was higher – 153,042 (22.56%) – than with the other scenarios: dental sealant: 141,097 (20.80%); supervised toothbrushing (STB): 128,655 (18.97%); base-case: 112,405 (16.57%). It increased by 36.2% under the combined STB+sealant, 25.5% in sealant, and 14.5% in STB compared to the base case. The untreated caries population was the smallest under combined STB+sealant – 257,655 (37.99%) – followed by sealants with 264,507 (38.99%), and STB with 271,467 (40.03%), but the base-case (no intervention) population group was the largest, with 280,244 (41.32%). Compared to the base case, it decreased by 8.1% in combined STB+sealant, 5.6% in sealant, and 3.1% in STB. The population with missing teeth and populations with restoration and endodontic treatment were also the largest in the base-case scenario, and the smallest with combined STB+sealant followed by sealant and STB. Compared to the base case, the population with restorations decreased by 6.1%, 4.4%, and 2.5% while the population with endodontic treatment decreased by 4.7%, 3.3%, and 1.9% in combined STB+sealant, sealant, and STB, respectively. The missing-teeth population decreased by 6.7% with combined STB+sealant, 4.8% with sealant, and 2.8% with STB compared to the base case.

**Table 1:** Simulation results when the age of 15 years

State	Scenarios			
	Base case (a)	STB (b)	Sealant (c)	STB + Sealant (d)
No caries	112,405 (16.57%)	128,655 (18.97%)	141,097 (20.80%)	153,042 (22.56%)
(% change from the base case)	–	+14.5	+25.5	+36.2
Untreated caries	280,244 (41.32%)	271,467 (40.03%)	264,507 (38.99%)	257,655 (37.99%)
(% change from the base case)	–	-3.1	-5.6	-8.1
Restoration	119,813 (17.67%)	116,808 (17.22%)	114,601 (16.90%)	112,547 (16.60%)
(% change from the base case)	–	-2.5	-4.4	-6.1
Endodontic	12,822 (1.89%)	12,574 (1.85%)	12,390 (1.83%)	12,222 (1.80%)
(% change from the base case)	–	-1.9	-3.3	-4.7
Missing teeth	152,959 (22.55%)	148,739 (21.93%)	145,648 (21.47%)	142,777 (21.05%)
(% change from the base case)	–	-2.8	-4.8	-6.7

STB (b), % change from the base case = (b – a)/ a x 100; sealant (c), % change from the base case = (c – a)/ a x 100; STB+Sealant (d), % change from the base case = (d – a)/ a x 100.

**Fig 3:**
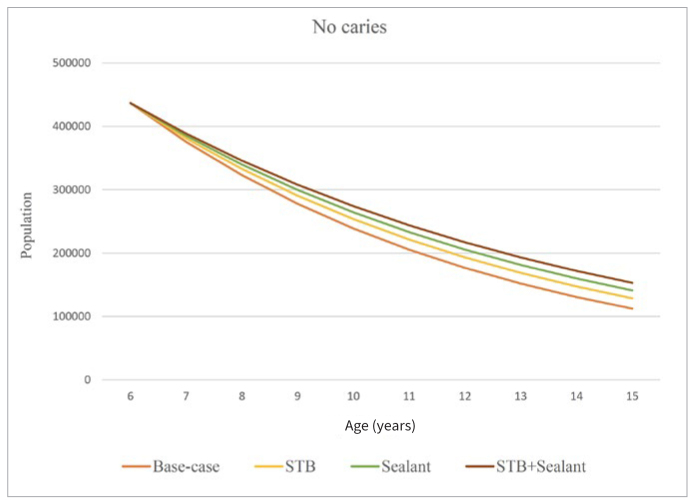
Caries-free population.

Figure 3 shows the permanent-dentition-age population with no caries (6 to 15 years old). The largest caries-free population was found under combined STB+sealant, followed by sealant and supervised toothbrushing (STB). The smallest caries-free population existed under the base-case scenario. Moreover, it is seen that the caries-free population under all scenarios gradually shrinks over time.

**Fig 4:**
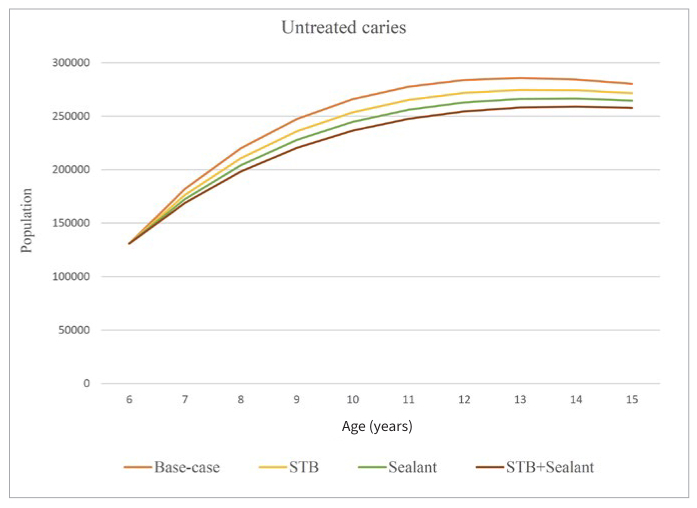
Population with untreated caries.

Figure 4 presents the untreated-caries population among 6- to 15-year-olds, the age group with at least partially permanent dentition. It is the smallest under combined STB+sealant, followed by sealant and STB. It is the highest given the base-case situation. Further, Fig 4 shows that the population with caries under all scenarios has slightly increased over the years.

The results of the sensitivity analysis showed the same trend. The mean values of the outcome (population with no caries) with a 95% confidence interval under the base-case scenario and intervention scenarios are presented in Table 2.

**Table 2:** Sensitivity analysis of the population with no caries at the age of 15 years

Scenarios	Population with no caries		
	Mean	Lower bound (95% CI)	Upper bound (95%CI)
Base-case	112,383	97,023	128,402
STB	128,592	112,815	144,913
Sealant	141,006	125,046	157,423
STB + sealant	153,526	137,157	169,079

## Discussion

Among the interventions administered by the Ministry of Public Health (MOPH) to the age range of 6 to 12 years, our results shown that the combination of STB and sealant is preferable to their application separately.

When establishing the model’s interventions, the efficacy rates of the interventions were identified and adjusted based on Thailand’s coverage rates. The efficacy rate of supervised toothbrushing (STB) was 9.25%, depending on the coverage rate. Although the coverage rate is high, the efficacy rate was not, and the rate of caries development was not substantially reduced compared to the baseline scenario. While the sealant and combined STB+sealant had superior efficacy rates, the coverage rates were unsatisfactory, and the adjusted efficacy rates were 15.7% and 19.9%, respectively. The authors suggest that the efficacy rates were not particularly high due to the relatively low coverage rates. Therefore, the reduction in caries development rate associated with these two interventions did not differ clinically significantly from the baseline.

Due to the unsatisfactory effectiveness rate of supervised toothbrushing (STB), this must receive greater emphasis in the intervention. A study found that STB has a positive influence on preventing caries.^16^ In this study, children participated in an intensive preventive programme that included the Bass method of brushing and finger flossing under the supervision of dentists. During the programme, daily brushing with fluoride toothpaste and flossing were practiced after lunch each school day for one semester (20 weeks) under the supervision of school nurses. The duration of this programme was approximately a decade. Consequently, the type of resource utilised and planning would be crucial for the intervention, and a comprehensive approach to toothbrushing should be considered for greater positive effects. In terms of sealants, applying resin-based sealants to the pits and fissures of permanent teeth has a positive effect on caries reduction. However, more resources would be required than for STB, and their availability should be a concern. Based on the simulation, the combination of STB and sealant is superior to administering each of these separately. Supervised toothbrushing (STB) is proposed not only to prevent the occurrence of caries but also to develop beneficial oral hygiene habits, whereas dental sealants are particularly effective at preventing caries on molars.^6,8^ The combination of the interventions therefore appears to be the most effective intervention. As shown in Table 1, for the populations of 15-year-olds with untreated caries, restorations, endodontic treatment, or missing teeth are smallest when the combined intervention is performed instead of each intervention administered alone.

Splieth et al^29^ developed the System Dynamics Model (SDM) with and without fluoride use and reported that the combination of fluoride toothpaste, fluoridated salt, and fluoride gel was the most cost-effective fluoride regime for caries prevention. Another study^13^ investigated the SDM for early childhood caries (ECC) interventions: applying fluorides, limiting bacterial transmission, utilising xylitol, clinical treatment, and motivational interviewing, and reported that the combined ECC interventions were the most effective method for caries reduction and costs. The present study is consistent with these previous studies in that SDM simulation indicates that combined interventions are more effective. In this investigation, targeting interventions for children ages 6 to 12 differs from previous research. In addition, previous studies only mentioned the effectiveness rates of the interventions, whereas this study considered both the effectiveness rates of the interventions determined by meta-analyses and the coverage rates in Thailand.

Consideration of each caries risk factor as a variable in the model is intricate, which is a limitation of the study. It is assumed that reducing risk factors, e.g., through various interventions, can prevent the development of caries. The majority of the data came from sources at the national level of Thailand, including the National Statistical Office, the National Oral Health Survey, the Health Data Center, and provincial-level literature which was reviewed by experts. However, there is a limitation of the employed database. Due to the lack of data at the national level for certain model parameters, the relevant literature was consulted to determine the values of some model parameters.

As another limitation, it is difficult to retrieve all data sources from the same year. Nevertheless, this would not detract from model validity, since the study is based on simulation and the model is run as a “what-if” scenario analysis to estimate the best scenario by inputting reliable data. In addition, the model was validated structurally and behaviourally by the experts mentioned above. For the coverage rates of interventions, the data were obtained from different years. On the other hand, if the coverage rates of interventions are subtracted from the same year, around 45% for STB and 13%-14% for sealant and combined STB+sealant, the simulation results of intervention scenarios would not differ from the base-case scenario.^5,11,22^ This is because the efficacy rates of interventions are low when the coverage rates are low, even though the interventions have a satisfactory effect on the outcome, since the efficacy rate is adjusted with the coverage rate. Therefore, the most favourable coverage rates of interventions from the different years were input into the model intended to determine the effect of interventions. It also shows that the coverage plays a role in improving the interventions’ efficacy rates.

Since it has been determined that a combination of STB and sealant is the most effective intervention, consideration must be given to increasing the intervention’s rate of coverage. This is due to the fact that after adjusting for the coverage rate, the efficacy rate of the combined intervention is quite low, despite the fact that the combined intervention is highly effective. According to previous studies conducted in Thailand, the unit cost of applying pit-and-fissure sealant per case was 243 baht, or $6.97, while the unit cost of oral hygiene instruction and an oral health examination was 109 baht, or $3.20.^27,32^ In light of the expanding coverage rate of interventions, therefore, the available resources are crucial.

The System Dynamics Model (SDM) can be used for long-term analysis of the effectiveness of interventions when predicting their impact on oral health. In addition, the SDM could be useful in contrasting cases as “what-if” scenarios in order to predict the best case and worst case of other services or policies. Since the study did not assess the costs, cost-effectiveness, or cost-savings, these aspects should be evaluated in future research, which should also consider the intervention resources.

## Conclusion

Among the interventions provided by the Ministry of Public Health (MOPH) for 6- to 12-year-olds, the combination of STB and sealant is more effective than each applied separately. The combined intervention has to be implemented taking the available resources into consideration. Moreover, the System Dynamics Model (SDM) would help decision-makers anticipate the best interventions or policies by simulating scenarios.
